# QDentalNet: a geometry-aware hierarchical mesh learning and quantum-enhanced network for automated dental occlusion classification

**DOI:** 10.3389/frai.2026.1931803

**Published:** 2026-10-21

**Authors:** Ramakrishnan Ilanghovan, S. Alden Jenish, R. Karthik, K. Suganthi, R. Praveen

**Affiliations:** 1 School of Computer Science and Engineering, Vellore Institute of Technology, Chennai, India; 2 School of Electronics Engineering, Vellore Institute of Technology, Chennai, India; 3 Centre for Cyber Physical Systems, Vellore Institute of Technology, Chennai, India; 4 Department of Computer Science and Engineering, National Institute of Technology, Puducherry, India

**Keywords:** convolutional neural network, deep learning, dental occlusion, mesh convolution, quantum computing

## Abstract

**Objectives:**

The accurate analysis of dental occlusion is important to orthodontic diagnosis and occlusal classification. The existing assessment procedures largely depend on clinician-driven evaluation of intraoral scans, which introduces subjectivity and limits scalability in routine diagnostic workflows. To address these challenges, this study proposes an automated dental occlusion classification architecture that integrates geometry-aware mesh learning with a quantum-enhanced network.

**Methods:**

The proposed QDentalNet employs a hierarchical mesh-processing backbone designed to capture both local and global geometric characteristics of dental structures. Rotation-aware feature encoding and kernel-based geometric correlation are incorporated through the Face Rotation Convolution (FRC) and Face Kernel Correlation (FKC) modules. The network also adopts a learning strategy with task towers and quantum-enhanced classification heads. This design helps the prediction of multiple clinically relevant occlusal classes. Additionally, auxiliary features via upper and lower contact point estimation enable efficient feature learning. The study also presents a novel composite loss function that enhances the discriminative capability of the network by addressing class imbalance, anatomical consistency, and efficient feature learning.

**Results:**

The proposed network achieved an average classification accuracy of 80.46% across all occlusal tasks. The obtained performance was also contextualized against previously reported results from existing 3D dental classification approaches as reference benchmarks.

**Conclusion:**

To the best of our knowledge, this is one of the initial studies to incorporate geometry-aware features with quantum-based decision-making for dental occlusion classification, providing a proof-of-concept for automated occlusal assessment.

## 1 Introduction

Dental occlusion refers to the way the maxillary (upper jaw) and mandibular (lower jaw) teeth contact during jaw closure and functional movements. It plays an important role in mastication, speech, facial aesthetics, temporomandibular joint functions, and long-term oral health. Malocclusion is defined as an abnormal relationship between the dental arches or between individual teeth. It is associated with increased dental caries risk, periodontal breakdown, traumatic occlusal contacts, temporomandibular joint disorders, and reduced quality of life (Leck et al., 2022). Epidemiological investigations report that clinically significant malocclusion affects a large proportion of children and adults worldwide. As a result, orthodontic diagnosis and treatment planning represent a major component of modern dental practice (Macey et al., 2020; Lombardo et al., 2020).

Clinically, occlusion is described along three principal dimensions. These include the sagittal dimension, which describes anteroposterior relationships. The vertical dimension describes overbite and open bite conditions. The transverse dimension describes crossbite and scissor bite relationships. The most common sagittal classification scheme is Angle's molar classification. Class I, Class II, and Class III relationships can be defined based on the anteroposterior positioning of the first molars and incisors. Vertical discrepancies such as deep bite and anterior open bite may coexist with transverse discrepancies, midline deviations, and underlying skeletal asymmetries. Consequently, three-dimensional assessment of the maxilla and mandible can provide clinically relevant information that may not be fully captured by conventional two-dimensional records. Conventional methods of diagnosis include visual examination, two-dimensional photography, dental study casts, and cephalometric radiographs. However, these have been shown to be limited in demonstrating actual 3D occlusal discrepancies (Rischen et al., 2013; Kapila et al., 2011).

Prior to the incorporation of Deep Learning (DL) techniques, computer-aided diagnosis in orthodontics primarily relied on geometric features designed by human experts. These geometric features were created from either a cephalometric radiograph or digital dental cast images. Typical characteristics that were used in this method of diagnosis included angular measurements, linear distances, arch form values, and points defined by the human expert. Additionally, conventional Machine Learning (ML) algorithms, including support vector machines, random forest classifiers, and k-nearest neighbors, were applied to these designed geometric characteristics to evaluate malocclusion diagnosis or prediction of future growth patterns. These approaches relied on handcrafted features derived from expert knowledge, limiting scalability and reducing the ability to generalize across diverse clinical scenarios. Additionally, the ML algorithms could not fully model the complexity of spatial structure when attempting to evaluate a three-dimensional representation of dental anatomy (Liu et al., 2021).

Advances in three-dimensional imaging technologies have enabled more detailed digital representations of the dentition and craniofacial structures. Cone-beam computed tomography and high-resolution intraoral or laboratory scanners are now widely used in orthodontic workflows (Mohammad-Rahimi et al., 2011). Concurrently, 3D DL architectures have been developed for voxel grids, point clouds, and surface meshes. These models enable end-to-end learning of hierarchical geometric features directly from raw three-dimensional data (Sivari et al., 2023; Wu et al., 2022). In dentistry, DL has been applied to tooth segmentation, jaw segmentation, lesion detection, and implant planning using 3D volumes or surface models. Several studies have reported performance comparable to expert annotations in specific dental imaging tasks. Despite this progress, most existing three-dimensional dental models process a single jaw independently or compress occlusion into global descriptors. Fine-grained contact relationships between the upper and lower arches are often not explicitly modeled. These relationships are vital for accurate occlusion assessment (Sivari et al., 2023).

Recent reviews of DL in dental imaging identify many open challenges. These include effective use of full three-dimensional surface information, robustness to small and imbalanced clinical datasets, and integration of multi-task outputs that reflect clinical reasoning (Liu et al., 2021; Mohammad-Rahimi et al., 2011; Sivari et al., 2023). Another challenge is including anatomical priors to limit unrealistic predictions. Mesh-based neural networks address some of these limitations. They perform directly on the surface meshes and use either a face- or edge-centered representation of the mesh to more efficiently model local curvature and shape compared to voxel-based or point-based methods (Wu et al., 2022). However, these architectures have primarily been evaluated on generic shape analysis benchmarks. Their application to orthodontic occlusion analysis remains limited. This creates a gap between advances in three-dimensional mesh learning and clinically grounded occlusion diagnosis using real dental datasets (Liu et al., 2021; Wu et al., 2022).

This study proposes a three-dimensional mesh-based neural network for automated occlusion assessment using paired maxillary and mandibular surface models. The method introduces a cross-jaw feature representation that encodes the complete occlusal environment based on the combination of location coordinates of face centers, surface normals, corner geometry, and explicit vectors of interaction between the two opposing arches. A task-specific attention mechanism is applied prior to global pooling to learn independent spatial focus maps for five clinically relevant outputs: right sagittal class, left sagittal class, anterior bite condition, transversal bite condition, and dental midline alignment. To improve the efficiency of the proposed network, a symmetric contact loss is incorporated to encourage bilateral consistency and realistic vertical alignment of maxillary and mandibular contact regions. In addition, the network integrates hybrid quantum-classical classifiers at the task-specific output stage, where low-dimensional embeddings are processed through variational quantum circuits and fused with classical pathways, enabling exploration of quantum-enhanced decision modeling while maintaining training stability and clinical relevance.

## 2 Related works

Growing literature in the field of medical image analysis highlights efficient DL architectures for dental classification and segmentation. While early computer-aided diagnostics relied on 2D radiographic imaging, recent advancements in DL have paved the way to more sophisticated volumetric analysis in this area of research.

Kim et al. (2023) introduced an automated method utilizing 2D CNN combined with dental CT data and 3D optical scans to segment the maxilla and mandible from projection images. The study employed a curvature variance of neighbor metric to effectively distinguish occlusal surfaces from smooth regions. Wu et al. (2015) introduced a convolutional deep belief network that represents 3D geometric shapes as probability distributions of binary variables on a 3D voxel grid. The model used single-view 2.5D depth maps and captured hierarchical part representations to perform joint object recognition and shape completion. Liu et al. (2016) implemented a dual-track network that utilizes spatio-temporal features from raw depth sequences. The study presented a 3D-based deep CNN to extract geometric and temporal patterns directly from depth maps, which are complemented with spatial relationships and angles. Dong et al. (2014) presented a convolution-free neural network that uses Multi-layer Perceptrons (MLP) instead of traditional convolutional modules. This design helped handle irregular triangular meshes by using high-level geometric features such as heat kernel signature and dihedral angles to effectively model complex and watertight manifolds. Zhu et al. (2022) implemented a local information fusion network that shifts focus from global view descriptors to localized feature extraction, unlike standard multi-view 3D recognition. The study introduces a region organization module to reorganize the feature maps into informative regions and utilizes attention mechanisms to model intricate relationships. Shi et al. (2025) presented a dual-module network to capture both local details and global context simultaneously. The model includes feature discrimination modules to isolate key local geometries, coupled with a context propagation module that adds global spatial information. Xia introduced a shape classification model that projects 3D geometry into dual-channel 2.5D thickness and depth views (Xia, 2023). By converting the views into a single 2D image, the model learns rich information about locality and topology features with reduced computational costs. Riegler et al. (2017) employed a specialized representation of a 3D DL model that enables high-resolution convolutional networks by exploiting data sparsity. The study partitions 3D space using unbalanced octrees and focuses memory and computation only on required dense regions.

Cui et al. (2021) presented a two-stage model using 3D point cloud data to tackle clinical irregularities such as missing or misaligned teeth. The study utilized a distance-aware centroid voting scheme for efficiently localizing tooth areas, followed by a confidence-aware cascade segmentation module. Bickel et al. (2022) implemented a method to retrieve geometries by converting surfaced meshes into point clouds, followed by feature-extracting matrices. This alignment process enables unsupervised similarity searches without requiring labeled data. Khodadad et al. (2024) presented a multi-level graph convolutional neural network for 3D point cloud analysis. This graph neural network coupled with K-nearest neighbor graphs captures multi-scale spatial features with minimal computational overhead, unlike conventional 3D architectures. The architecture achieved a thousandfold reduction in floating-point operations and significant savings of storage while maintaining improved performance. Wang et al. (2025) introduced a self-supervised representation learning model that learns global semantic understanding with local geometric reconstruction. The study employed a cross-modal mask alignment process and mapping between 3D point clouds and 2D image patches. This ensures information symmetry is maintained across modalities while enhancing multi-scale feature encoding. Ye et al. (2025) employed a local-non-local complementary learning network to address the limitation of unstructured point cloud data. The study utilizes partial convolution to separate feature extraction into distinct local and non-local branches. The study also employs a central complementary interactive attention module that adaptively fuses these features, which allows a balanced representation against noise and sparsity.

Liu and Zhao (2024) utilized an enhanced MeshNet architecture to address the classification difficulties across different 3D classes. The study replaced standard cross-entropy loss with combined focal and regularization loss to effectively reweight the model's focus toward harder samples while preventing overfitting. Mouncif et al. (2025) introduced a DL classification network for sequential analysis by addressing 3D data irregularity by converting 3D triangular mesh features into 2D grayscale images. The study extracted vertex coordinates and triangle centers as time-influenced sequences, and an RNN was used to capture spatial relationships within the dental anatomy. Shu et al. (2025) presented a transformer-based model for 3D meshes that learns intrinsic manifold information such as dihedral angles and geodesic distances. To address the limitation of standard 3D representations, the study developed a face walk serialization algorithm that changes the geometry and topology of the mesh into sequences of tokens. This helps capture local and global features while preserving structural and spatial relationships. Chen et al. (2022) employed a hierarchical 3D mesh transformer coupled with the self-attention mechanism of NLP to model unstructured 3D surfaces. The study partitions mesh models into shape and visual tokens for high-level feature summarization. The Poisson sampling was used to create a multi-resolution pyramid structure and a vector-type self-attention mechanism to learn local geometric context and global semantic structure. Liang et al. (2024) introduced a contrastive learning network designed for 3D manifold mesh analysis to avoid the limitations of small, labeled datasets. The method maximizes similarity between positive pairs generated through mesh-specific augmentations and minimizes it against negative pairs by regularized learned representations. Gao et al. (2022) leverage a graph convolutional neural network that processes triangular meshes by treating each triangular face as a discrete unit. The study utilizes an adjacency matrix from the mesh topology to analyze the non-Euclidean relationships between neighboring faces.

In summary, the advancement of DL and geometric modeling has transformed the classification of 3D structures beyond conventional 2D diagnostics toward efficient volumetric analysis. By preserving the intrinsic topology and inter-arch spatial dynamics of intraoral scans, these evolving techniques pave the way for more precise and scalable solutions in occlusal classification.

### 2.1 Research gaps

Although extensive progress has been made in three-dimensional dental analysis, few limitations remain relevant to automated multi-attribute occlusal classification. The gaps are as follows:

- Existing approaches include individual-arch shape as well as geometric and contact-based analysis using opposing dental arches. Paired-arch representation is vital since occlusion is inherently determined by the spatial relationship between the maxillary and mandibular dentitions. However, there remains limited research that combines explicit inter-arch geometric relationships directly with mesh representations for analysis of multiple occlusal attributes.
- Different occlusal characteristics depend on different anatomical regions and geometric relationships. They do not necessarily rely on identical spatial evidence. Single globally pooled architectures may therefore provide limited task-specific localization. A multi-task framework may provide a more appropriate representation for efficient performance.
- The integration of alternative classification mechanisms with geometry-aware mesh representations remains relatively underexplored in automated dental occlusion analysis. Hybrid quantum-classical classification provides an opportunity to investigate the feasibility of variational quantum circuits as task-specific decision modules within an otherwise classical DL framework.

### 2.2 Research contributions

The proposed network incorporates a hybrid network to enhance 3D dental model analysis. The contributions include:

- The model incorporates a specialized 3D mesh processing pipeline that integrates structural features such as face centers, normals, and corners to represent the full occlusal environment with interaction vectors between the upper and lower arches.
- The architectures employed a multidimensional attention module that generates independent attention maps for five distinct clinical tasks. This helps the model to focus on the fine-grained regions that are relevant to specific diagnoses before feature pooling.
- The network incorporates a hybrid quantum-classical classification stage in which low-dimensional task-specific embeddings are processed using Variational Quantum Circuits (VQCs). This component is introduced to investigate the feasibility of integrating parameterized quantum circuits within the task-specific classification stage of a geometry-aware dental mesh network.
- The proposed network also incorporates a symmetry contact loss function. This enforces geometric consistency and occlusal contact point constraints, which help the model's internal representations with respect to the physical anatomy of the human jaw.

## 3 Proposed network

The proposed QDentalNet design is an end-to-end learning system that performs automated multi-attribute occlusal classification directly from 3D intraoral scans. Unlike conventional methods that rely on 2D projections, this system leverages raw mesh data to preserve the intricate geometric topology of the dental arches. The preprocessing aligns raw upper and lower STL models within a common coordinate system to compute inter-arch interaction vectors. These vectors are subsequently analyzed by the 3D MeshNet backbone together with cyclically invariant, rotation-aware FRC feature encoding and an attention mechanism that prioritizes task-relevant anatomical regions. The network also incorporates hybrid quantum-classical classification heads, in which low-dimensional task-specific embeddings are processed using parameterized variational quantum circuits before final classification. The VQC component is evaluated as a proof-of-concept hybrid decision module rather than as evidence of improved representational capacity or quantum advantage. The global optimization is modeled by a composite loss function that synergizes weighted cross-entropy with a symmetry-contact loss function. Figure 1 illustrates the architectural overview of the proposed network.

**Figure 1:**
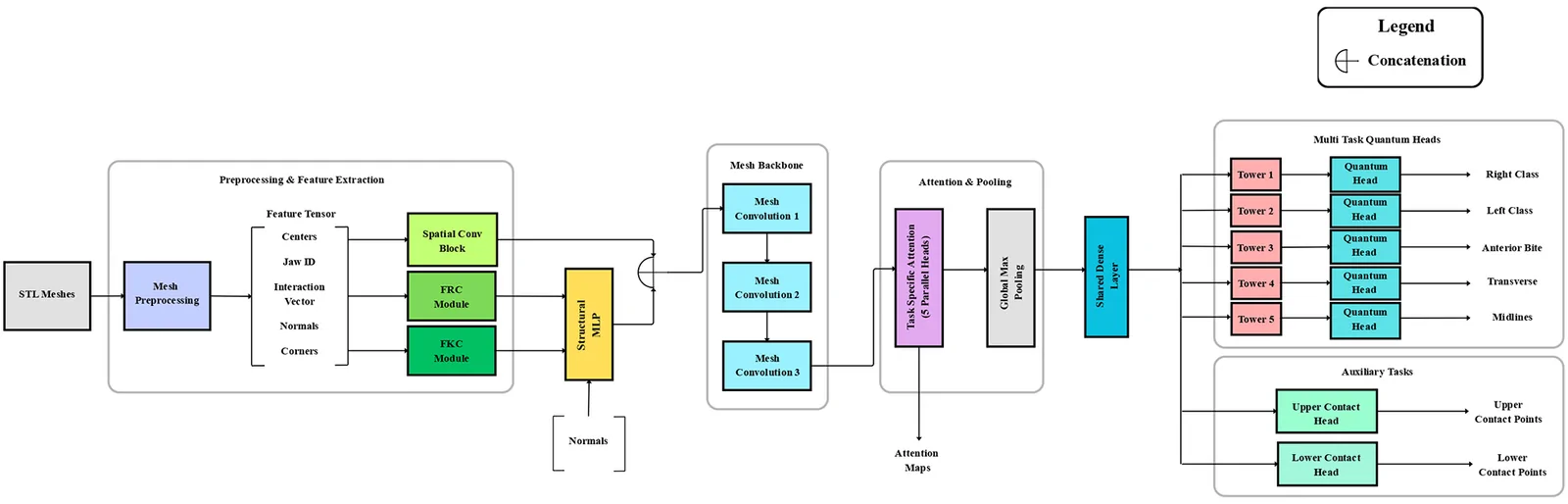
Overall architecture of QDentalNet. The network consists of cross-jaw feature extraction, FRC, FKC, hierarchical mesh-convolution blocks, task-specific attention, auxiliary contact-point prediction heads, and quantum-enhanced classification heads for multi-task occlusal diagnosis.

### 3.1 Cross-jaw feature extraction

The cross-jaw feature extraction module is designed to encode the occlusal environment by jointly modeling the geometric and spatial relationships between the upper and the lower meshes. Unlike conventional approaches that process each arch independently, the proposed module integrated both jaws within a unified geometric representation to explicitly capture inter-arch contact dynamics and 3D spatial dependencies. The overall workflow of the module is illustrated in Figure 2. Initially, the upper and lower STL meshes are transformed into a common coordinate system to preserve their true occlusal alignment. To reduce computational overhead, the random sampling mechanism is applied to both the arches. To ensure computational efficiency, each mesh was uniformly downsampled from an original average of approximately 182,444 faces to 8,192 triangular faces prior to feature extraction.

**Figure 2:**
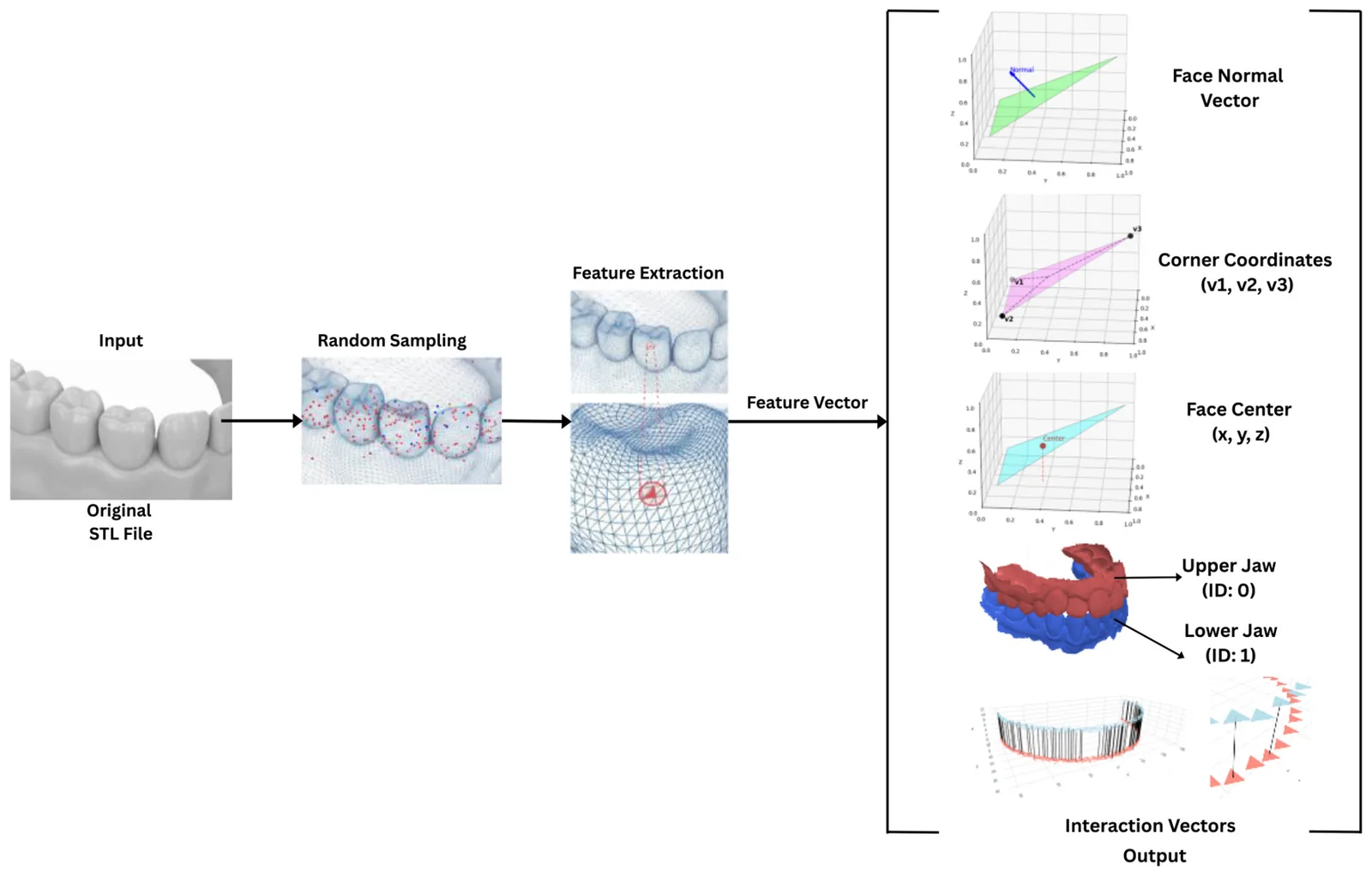
Workflow of the cross-jaw feature extraction module, including mesh sampling, extraction of face centers, normals, corner coordinates, and computation of upper-lower interaction vectors.

To model inter-arch relationships, the module computes interaction vectors between spatially corresponding regions of the upper and lower jaws. These vectors are constructed between face centers of opposing arches and encode both directional and magnitude-based information. This helps represent vertical clearance, sagittal displacement, transverse asymmetry, and potential contact tendencies. The magnitude of the interaction vector reflects inter-arch separation, while its direction captures the relative orientation of occluding surfaces. Correspondence between the upper and lower arches was established using nearest-neighbor matching between face centers. For every sampled face center in the maxillary arch, the closest face center in the mandibular arch was identified using Euclidean distance within a spatial search structure. The interaction vector was subsequently computed as the displacement between the paired face centers. This procedure enables the network to model vertical separation, transverse displacement, and sagittal relationships between opposing dental surfaces.

### 3.2 Face rotation convolution

The Face Rotation Convolution (FRC) module is designed to reduce sensitivity to the arbitrary ordering of the vertices used to represent each triangular mesh face (Feng et al., 2019). For every sampled face, the module receives a corner-based representation derived from the coordinates of the triangle vertices. Because the starting vertex assigned during mesh triangulation is arbitrary, equivalent geometric faces may be represented by different cyclic orderings of the same vertices. To address this representation ambiguity, the FRC constructs three feature streams corresponding to cyclic permutations of the triangle-vertex ordering. Each stream is processed using a shared point-wise Multi-Layer Perceptron (MLP), and the resulting representations are combined using element-wise averaging. Let (*g*(*x*)) denote the shared feature transformation applied to a triangle representation (*x*) and let (P) denote the cyclic permutation operator on its vertex ordering. The FRC aggregation can be expressed as in Equation 1.


$$\begin{array}{l} h\left(x\right)=\frac{1}{3}\left[g\left(x\right)+g\left(Px\right)+g\left(P^{2}x\right)\right] \end{array}$$ (1)

Applying an additional cyclic permutation to the input only reorders the three terms in the summation. Therefore, *h*(*Px*) = *h*(*x*) establishes invariance of the aggregated representation to cyclic changes in triangle-vertex ordering, as per Equation 1. This operation does not constitute mathematical invariance to arbitrary rigid-body rotations of the complete three-dimensional mesh. A global rotation transforms the underlying coordinate values and may therefore modify the learned coordinate-based representation. The study distinguishes this cyclic index invariance from geometric invariance under global rigid-body rotations of the complete mesh. Because the module directly processes coordinate-based features, global transformations modify the underlying spatial values. Consequently, the FRC functions as a cyclically invariant and rotation-aware feature extractor rather than an intrinsically rotation-invariant operator.

In summary, the FRC performs three complementary operations: point-wise transformation of corner-coordinate features, cyclic symmetrization across equivalent triangle-vertex orderings, and feature fusion through a shared MLP. The cyclic averaging operation explicitly removes dependence on the arbitrary starting vertex used during triangular-face representation. The architectural overview of the FRC module is illustrated in Figure 3.

**Figure 3:**
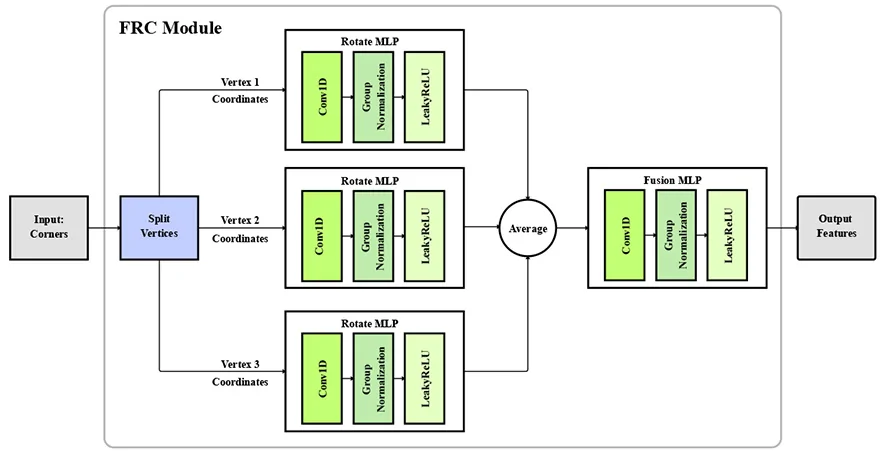
Detailed architecture of the FRC module illustrating vertex decomposition, rotation-aware MLP processing, feature averaging, and fusion.

### 3.3 Face kernel correlation

The Face Kernel Correlation (FKC) module is designed to characterize local variations in surface orientation by comparing face-normal vectors with a set of learnable directional kernels. The neighborhood supplied to the FKC is defined spatially rather than in normal space or directly from mesh connectivity. Specifically, after mesh down sampling, the center of each sampled triangular face is computed in the normalized coordinate system. For each face, the (*K*_*intra*_ = 10) nearest faces from the same dental arch are identified according to Euclidean distance between face centers. In addition, (*K*_*cross*_ = 2) nearest faces from the opposing arch are identified using the same face-center Euclidean distance criterion. Consequently, each face is associated with a spatial neighborhood containing local same-arch faces together with a small number of geometrically proximal cross-arch faces.

Let $n_{i}\in R^{⊯}$ denote the unit normal of the *i*-th face and let N(i) represent its spatially defined neighborhood. While the FKC does not compute differential curvature directly, the angular deviations among adjacent unit normals yield an effective representation of local surface topography. To maintain precision, the study designates this metric as normal-orientation variation rather than differential curvature. Cross-jaw proximity is established before the FKC through nearest-neighbor relationships between face centers, while explicit inter-arch displacement is represented separately by the cross-jaw interaction vectors. The FKC can incorporate the normal orientations of spatially proximal opposing faces through the cross-jaw neighbors, but the complete model combines these orientation descriptors with the explicit spatial and interaction features to represent occlusal relationships. To learn orientation-dependent geometric patterns, the FKC employs a set of learnable directional kernels. For each FKC channel (*m*), four directional components are parameterized by two trainable angular variables, α_*m, r*_ and β_*m, r*_ where *r* denotes the directional component. The corresponding kernel direction is defined as in Equation 2.


$$\begin{array}{l} \left[\begin{array}{c} \sin\left(\alpha_{\left(m,r\right)}\right)\cos\left(\beta_{\left(m,r\right)}\right)\\ \sin\left(\alpha_{\left(m,r\right)}\right)\sin\left(\beta_{\left(m,r\right)}\right)\cos\left(\alpha_{\left(m,r\right)}\right) \end{array}\right] \end{array}$$ (2)

This spherical-coordinate parameterization constrains every learned kernel vector to unit norm. The formulation is therefore consistent with the unit-normal representation of the mesh faces and allows the learned kernels to represent reference directions on the unit sphere. In the implemented network, the FKC contains 48 output channels, with four learnable directional components associated with each channel. For each local face normal *n*_*j*_, where j∈N(i) its similarity to a learnable kernel direction is determined from the squared Euclidean distance $|w_{m,r}{|}^{2}$ where *m* indexes the FKC channel and *r* indexes the directional component of the corresponding kernel. The squared distance is subsequently converted into a Gaussian correlation response as give in Equation 3.


$$\begin{array}{l} \exp\left(-\frac{d_{ijmr}^{2}}{2\sigma^{2}}\right) \end{array}$$ (3)

where σ controls the width of the Gaussian response. The squared Euclidean distance is computed only once and is used directly in the Gaussian exponent. Thus, the formulation corresponds to the conventional Gaussian radial-basis response and does not involve a fourth-power distance. The Gaussian response increases when an observed face normal is closely aligned with a learned directional kernel and decreases as the orientation difference increases. For each FKC channel, correlation responses are aggregated across the center and neighboring face normals and across the four directional components associated with the kernel. This aggregation produces a compact descriptor indicating how strongly the local normal configuration corresponds to each learned orientation pattern. The resulting correlation features are subsequently normalized and passed through a non-linear activation before being forwarded to the downstream structural feature-fusion stage.

Through this mechanism, the FKC provides a learnable representation of local surface-orientation patterns that extends beyond the direct use of individual face normals. The module does not explicitly estimate surface curvature or independently determine inter-arch contact. Instead, it complements the raw normal features by characterizing the orientation distribution of spatially neighboring surfaces, including proximal faces from the opposing dental arch. These learned orientation descriptors are subsequently combined with the FRC features, raw geometric information, and explicit cross-jaw interaction features, enabling the downstream mesh-processing network to jointly model local surface geometry and inter-arch spatial relationships. The architectural overview of the FKC is illustrated in Figure 4.

**Figure 4:**
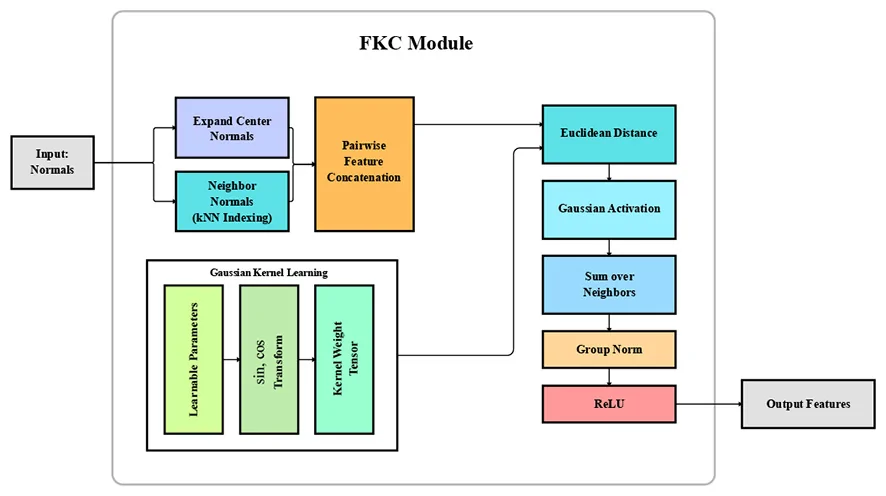
Detailed architecture of the FKC module showing neighborhood construction, Gaussian kernel correlation, aggregation, and feature generation.

### 3.4 Loss functions

The proposed architecture was trained using a novel composite loss function to improve geometric learning and classification performance. The loss function is designed to address the challenges of class imbalance, anatomical consistency, and efficient feature learning within the dental meshes. The network integrates weighted cross-entropy loss representing the loss from occlusion classification as part of the network, a symmetric contact loss that enforces geometric relationships between the upper and lower arches, and an attention sparsity loss to model the network to focus on the key regions of interest in the dental meshes. The final objective function is formulated as the weighted sum of these loss components, which enable the network to jointly learn discriminative and anatomically meaningful representations for efficient occlusion analysis.

#### 3.4.1 Weighted cross-entropy loss

To address the inherent class imbalance that is present in the dataset utilized, the proposed system employs a Weighted Cross-Entropy Loss (*L*_*CE*_) with label smoothing for each *k* classification task. This ensures that underrepresented occlusal classes also contribute equally during the training, thereby preventing the model from getting biased toward dominant classes. For a smoothing coefficient ϵ the smoothed target variable is given by Equation 4. The weighting scheme is implemented using inverse-frequency class weights, which increase the penalty associated with misclassification of rare categories. The loss function is represented by Equation 5, where *C*_*k*_ represents the number of classes for the task *k*, *w*_*c*_ represents class weight, *y*_*c*_ represents the ground-truth label, and *p*_*c*_ represents the predicted probability for the class *c*.


$$\begin{array}{l} y_{c}^{LS}=\left(1-\epsilon \right)y_{c}+\frac{\epsilon }{C_{k}} \end{array}$$ (4)

$$\begin{array}{l} L_{CE}^{\left(k\right)}=-\overset{C_{k}}{\underset{c=1}{\sum }}w_{c}y_{c}^{LS}\log\left(p_{c}\right)\; \end{array}$$ (5)

#### 3.4.2 Symmetric contact loss

To ensure anatomically consistent contact prediction, the network incorporates a Symmetric Contact Loss (LSC) composed of two important constraints: an occlusion constraint and a bilateral symmetry constraint. These components collectively force the model to learn realistic inter-arch alignment and structural symmetry within the predicted contact points. The symmetric contact loss is given by Equation 6.


$$\begin{array}{l} L_{SC}=L_{occ}+L_{sym} \end{array}$$ (6)

This regularization helps suppress physically implausible contact configurations while preserving the dominance of classification losses for target diagnostic tasks. Crucially, the bilateral constraint does not enforce strict anatomical symmetry across the predicted occlusal relationships. It operates instead as a soft prior over the auxiliary contact-point descriptors. Given that clinically diagnostic asymmetries frequently characterize conditions such as dental midline shifts and unilateral crossbites, the contribution of this penalty is governed by a dedicated weighting coefficient within the aggregate objective and validated through class-specific ablation studies.

#### 3.4.3 Occlusion loss

The Occlusion Loss (*L*_*occ*_) is designed to simulate near-realistic vertical bite interaction by minimizing the mean Euclidean distance between corresponding predicted contact points on the upper $\left({\hat{C}}_{m}^{U}\right)$, lower $\left({\hat{C}}_{m}^{L}\right)$ dental arches, and *N*_*c*_ sample count, given by Equation 7. This acts as a geometric cohesion constraint that encourages physically possible contact alignment between opposing surfaces. By reducing the spatial discrepancy, the model learns to produce contact predictions that reflect efficient occlusal relationships.


$$\begin{array}{l} L_{occ}=\frac{1}{N_{c}}\overset{N_{c}}{\underset{m=1}{\sum }}\;||{Ĉ}_{m}^{U}-{Ĉ}_{m}^{L}\;|{|}_{2} \end{array}$$ (7)

#### 3.4.4 Symmetry loss

The Symmetry Loss (*L*_*sym*_) is introduced to penalize deviations between predicted contact points on the left side (${Ĉ}_{m}^{L}$) of the dental arch and the sagittal reflection of the corresponding points on the right side ($\left({\hat{C}}_{m}^{R}\right)$, given in Equation 8. The reflection operator (Φ) performs transformation by negating the *x*-coordinate of the right-side points to effectively mirror them across the sagittal plane. Minimizing this term helps the model preserve the natural bilateral symmetry of the human jaw structure.


$$\begin{array}{l} L_{sym}=\frac{1}{N_{c}/2}\overset{N_{c}/2}{\underset{m=1}{\sum }}\;||{Ĉ}_{m}^{L}-\Phi \left({Ĉ}_{m}^{R}\right)\;|{|}_{2} \end{array}$$ (8)

#### 3.4.5 Attention sparsity loss

The Attention Sparsity Loss (*L*_*att*_) function is incorporated to enable the network to focus on relevant anatomical regions rather than relying on diffuse global representations. The function employs an L1 regularization penalty to the attention maps *A*_*k*_, generated for each task *N*_*t*_ with respect to the total sample count *N*. The sparsity constraint enables the attention mechanism to produce concentrated activations that highlight the most discriminative occlusal regions. The formulation of the loss function is given in Equation 9.


$$\begin{array}{l} L_{att}=\overset{N_{t}}{\underset{k=1}{\sum }}\overset{N}{\underset{i=1}{\sum }}\;||A_{k}\left(i\right)\;|| \end{array}$$ (9)

#### 3.4.6 Final objective function

The final loss function combines all loss components as a weighted sum as given by Equation 10, where λ_*k*_ represents task-specific loss weights and β and α are the global regularization coefficients. The combined objective enables the proposed network to simultaneously optimize classification accuracy, anatomical consistency, and feature localization. In all experiments, equal weighting was assigned to the classification losses of the individual tasks (λ_*k*_ = 1.0). The regularization coefficients for the symmetric contact loss and attention sparsity loss were empirically selected through preliminary validation experiments and set to β = 0.02 and γ = 10^−6^, respectively. These values provided a suitable balance between classification performance and anatomical consistency during training.


$$\begin{array}{l} L_{total}=\overset{K}{\underset{k=1}{\sum }}\lambda_{k}L_{CE}^{\left(k\right)}+\beta L_{SC}+\gamma L_{att} \end{array}$$ (10)

### 3.5 Quantum heads and final classification

The final classification layer employed a quantum mechanism to jointly predict multiple occlusal attributes while leveraging shared geometric representations learned by the proposed network. The output features from the backbone are forwarded into five parallel task-specific towers, each consisting of lightweight feature adaptation layers that project the shared mesh embedding into relevant latent space. Each task tower consists of a lightweight MLP that performs feature adaptation and transforms the shared embeddings into a latent embedding *z*_*k*_. These latent embeddings are then forwarded into a VQC classification head, which enables hybrid-quantum decision modeling. Each latent embedding *z*_*k*_ is first projected into an *n*_*q*_ dimensional pre-quantum feature space using a batch-normalized layer. The resulting vector is encoded into a quantum state using angle encoding, where each feature controls the rotation of a corresponding qubit. This encoding allows the classical features to be represented within the quantum state that can be subsequently processed by the circuit. Each task-specific classifier employed a variational quantum circuit consisting of six qubits and three trainable variational layers. Prior to quantum processing, the latent feature representation was projected into a six-dimensional feature vector through a batch-normalized linear layer. The projected features were encoded into the quantum state using angle embedding, where each feature controlled the rotation angle of a corresponding qubit. The encoded quantum states were subsequently processed using strongly entangling layers composed of parameterized single-qubit rotations and entanglement operations. The expectation value of the Pauli-Z operator from each qubit was measured and forwarded to the final classification layer.

The quantum classification component was implemented using PennyLane with the PyTorch interface. Each task-specific classifier employed a variational quantum circuit consisting of six qubits and trainable variational layers. The expectation value of the Pauli-Z operator was measured for each qubit and mapped to the corresponding task logits through a classical output layer. All reported quantum-head experiments were conducted using the same simulator/backend configuration to ensure reproducibility.

In addition to predicting occlusal points on both the upper and lower dental arches, the network uses auxiliary tasks to predict contact point locations for both arches. The two auxiliary heads are connected to shared backbone features and are trained to classify occlusal points on their respective arches, providing geometric guidance to the model and helping it learn anatomically meaningful representations. The auxiliary tasks also act as regularizers that reduce overfitting and promote consistent feature learning. The total loss calculated during training represents a weighted sum of all the individual class losses along with that of the auxiliary task, which provides equal optimization between all four objectives. Overall, the layer enables prediction of multiple occlusal attributes within a unified network.

## 4 Results and discussion

This section presents the overview of the dataset utilized, data augmentation techniques applied, and other details such as environmental setup and hyperparameter tuning employed. Additionally, ablation experiments are conducted to evaluate the specific contributions of various components within the network to its overall performance.

### 4.1 Dataset description

The Bits2Bites dataset used in this research consists of 200 paired, registered intraoral scans provided in STL format (Borghi et al., 2025). It comprises high-resolution meshes of the upper and lower dental arches, spatially aligned to preserve the true occlusal relationship. The data were acquired using two intraoral scanners to capture inter-device variability, with scans randomly selected to reflect real-world clinical diversity. Each pair was annotated by an experienced orthodontic specialist and included comprehensive multi-label occlusion annotations containing sagittal, transversal, and midline alignment categories. On average, each mesh contains 92,201 ± 28,140 vertices and 182,444 ± 55,862 faces, with approximate bounding-box dimensions of 65.9 ± 4.12 mm width, 53.84 ± 4.5 mm depth, and 17.9 ± 1.9 mm height, and a mean surface area of 3,780 ± 409 mm^2^. The five classification tasks correspond to clinically defined occlusal characteristics in the Bits2Bites annotation protocol: right and left sagittal relationships based on Angle's classification, anterior bite condition, transverse relationship, and dental midline alignment.

The dataset was partitioned into mutually exclusive training, validation, and test subsets. The training subset was used for optimization of the network parameters, while the validation subset was used for hyperparameter selection, learning-rate scheduling, and selection of the best-performing model checkpoint. The test subset remained isolated from all training and model-selection procedures and was accessed only after the architecture and training configuration had been finalized. Figure 5 illustrates sample STL images from the dataset. It should be noted that the dataset employs a multi-label annotation protocol in which each scan pair receives independent labels across multiple occlusal dimensions. The class frequencies reported in Table 1 correspond to the original task-specific annotations provided with the dataset and therefore do not represent a single mutually exclusive partition of the 200 scan pairs.

**Figure 5:**
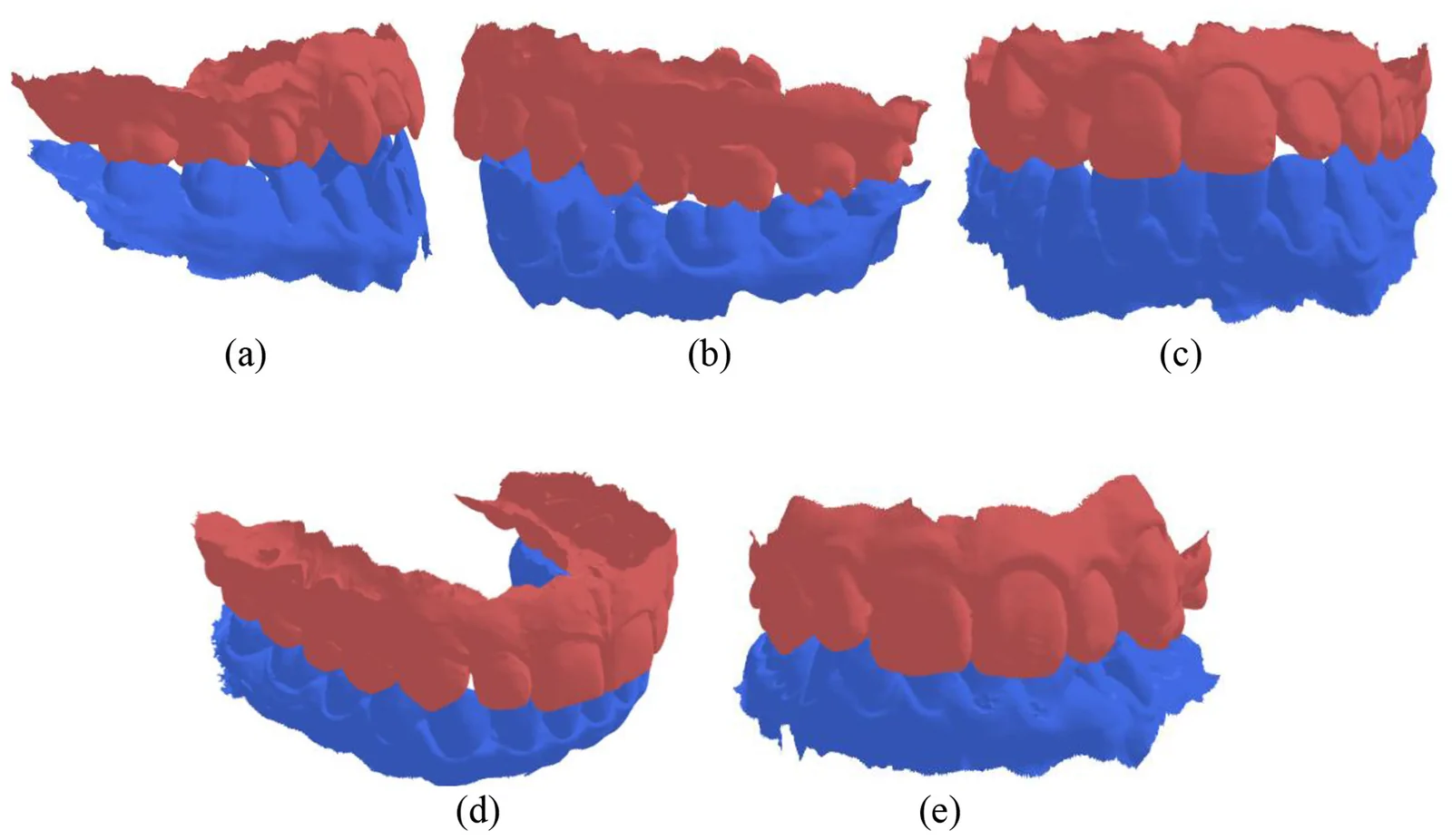
Sample dental STL images from the Bits2Bites dataset: **(a)** sagittal right, **(b)** sagittal left, **(c)** anterior bite, **(d)** transversal bite, and **(e)** midlines.

**Table 1:** Summary of distribution of the dataset classes produced from the Bits2Bites dataset.

Classes	Tasks	Number of samples	Train set (before augmentation)	Train set (after augmentation)	Validation set	Test set
Sagittal right	Class I	90	126	684	15	32
	Class II	6	2	33	2	2
	Class III	21	12	253	3	6
Sagittal left	Class I	88	122	648	13	32
	Class II	5	1	24	2	2
	Class III	28	17	298	5	5
Anterior bite	Deep bite	74	53	713	6	14
	Normal	81	59	533	7	15
	Open bite	42	28	224	5	9
	Reverse bite	4	1	24	2	2
Transversal bite	Normal	140	37	241	7	12
	Crossbite	56	103	429	11	26
	Scissor bite	5	2	20	2	2
Midlines	Centered	78	55	339	6	17
	Deviated	122	85	431	14	23

### 4.2 Data augmentation

This section presents the augmentation methods employed in this research to improve model generalization and efficiency when learning from high-resolution 3D intraoral scans. Since the dataset size was limited and the data consists of high geometric fidelity meshes, data augmentation was applied to the training set to increase variability while preserving meaningful occlusal relationships. These augmentations help reduce overfitting and enable the model to learn robust geometric representations from diverse mesh configurations. The augmentation strategies used in this study include: (1) Random rigid rotations around the three anatomical axes to simulate variations in scanner orientation and patient positioning, (2) Uniform scaling within a small range to account for inter-subject anatomical size variability, and (3) Controlled geometric perturbations such as random vertex jittering and low-magnitude Gaussian noise applied to vertex coordinates to simulate minor mesh irregularities. All transformations were applied identically to both the upper and lower arches to maintain the true occlusal relationship between the jaws.

Data augmentation was primarily applied to the training subset. For extremely underrepresented classes for which an adequate number of original samples could not be retained in the validation subset, a limited number of augmented samples derived exclusively from the corresponding training-class samples were included in the validation subset to maintain class representation during model selection. No augmented samples were included in the held-out test subset, which consisted exclusively of original scan pairs and remained isolated from training and model-selection procedures. A summary of the dataset is presented in Table 1.

### 4.3 Environmental setup

All experiments were conducted using PyTorch on two NVIDIA Tesla T4 GPUs with 16 GB memory each. The network was optimized using the AdamW optimizer. Separate learning rates were employed for the classical and quantum components, with learning rates of 1 × 10^−4^ and 5 × 10^6^, respectively. The classical parameters used a weight decay coefficient of 1 × 10^−3^, while no weight decay was applied to the quantum circuit parameters. A ReduceLROnPlateau scheduler monitored the validation F1-score and reduced the learning rate by a factor of 0.5 after five epochs without improvement. The network was trained for 250 epochs. Model selection was based on the validation performance monitored throughout training, while the ReduceLROnPlateau scheduler adjusted the learning rate when performance improvements plateaued.

### 4.4 Hyperparameter tuning

Tuning the hyperparameters is an important step to maximize the learning capacity of the DL model. In this study, five main hyperparameters influencing training stability and model performance were optimized: (1) Learning Rate, (2) Weight Decay, (3) Optimizer Type, and (4) Batch Size. To control overfitting and maintain smoother model weights, weight decay regularization was applied with values in the range of 0.1–0.00001. The learning rate varied between 0.1 and 0.00001 to balance convergence speed and stability. Batch sizes of 8, 16, and 32 were tested. All hyperparameter optimization and model-selection decisions were performed exclusively using the training and validation subsets. The held-out test subset was not used to select the learning rate, weight decay, optimizer, batch size, loss coefficients, network configuration, or training epoch. During training, model checkpoints were selected according to validation-set performance, and the selected configuration was subsequently evaluated on the test subset only for final performance estimation. After completion of the hold-out experiment, the same finalized architecture and hyperparameter configuration was used for the five-fold cross-validation analysis without further tuning based on cross-validation performance. This separation ensured that information from the hold-out test samples did not influence model optimization or selection. The summary of the results is given in Table 2.

**Table 2:** Summary of hyperparameter tuning results.

Hyperparameter	Values range	Optimal
Learning rate	[0.1, 0.00001]	0.0001
Weight decay	[0.1, 0.00001]	0.001
Optimizer	[ADAM, SGD, RMSProp]	ADAM
Batch size	[8, 16, 32]	16

### 4.5 Ablation studies

This section presents the set of ablation studies conducted in this study to evaluate the contribution of individual components to the overall performance of the proposed dental occlusion classification. The configurations tested in the study were: (1) Proposed network without the FRC module, (2) Proposed network without the FKC module, (3) Proposed network with classical classification head, and (4) Proposed network.

#### 4.5.1 Analysis of proposed network without cross-jaw interaction features

This ablation experiment was conducted to evaluate the contribution of the cross-jaw interaction vectors to the overall performance of the proposed network. In this configuration, the interaction vectors computed between corresponding regions of the maxillary and mandibular arches were removed by setting the upper and lower interaction features to zero. Consequently, the network relied solely on local geometric descriptors, including face centers, normals, corner coordinates, and jaw identifiers, without explicit information regarding inter-arch spatial relationships. The absence of cross-jaw interaction features limits the model's ability to capture anatomical relationships between opposing dental arches, which are essential for identifying sagittal discrepancies, transverse asymmetries, and occlusal contact characteristics. The network achieved accuracies of 62.04%, 49.75%, 54.75%, 73.72%, and 56.00% for sagittal left, sagittal right, anterior bite, transversal bite, and midline classification, respectively, resulting in an overall accuracy of 59.25%. These findings indicate that the cross-jaw feature representation provides valuable contextual information regarding inter-arch relationships and contributes substantially to the discriminative capability of the proposed architecture.

#### 4.5.2 Analysis of the proposed network without the FRC module

In this experiment, the FRC module is removed from the proposed system, and the input corner-based features are forwarded directly to the geometric processing stages. This helps assess the importance of rotation-aware features and vertex-level consolidation in learning efficient geometric representations. Experimental results indicate the marginal difference in performance, particularly in sagittal and transversal occlusal tasks. The network achieved an average accuracy of 59.99% in the sagittal left class. The absence of the FRC module removes the cyclic symmetrization of triangle-vertex representations and the associated rotation-aware feature processing, which may reduce the consistency of corner-based geometric encoding. The observed performance difference therefore supports the contribution of the FRC to geometric feature representation, without implying invariance to arbitrary rigid-body rotations of the complete mesh.

#### 4.5.3 Analysis of the proposed network without the FKC module

This ablation experiment is tested to assess the importance of the FKC module while retaining the other components of the network. Without the FKC module, the network lacks explicit kernel-based modeling of local surface normal interactions. The observed results highlight the decline in occlusal classes such as anterior bite and midline with an accuracy of 68.45% and 55.33%, respectively. These results indicate that there are localized geometric relationships and curvature representations that cannot be fully represented by the standard aggregation techniques.

#### 4.5.4 Analysis of the proposed network without task-specific attention

This experiment evaluates the importance of the task-specific attention mechanism incorporated within the proposed architecture. In this configuration, the task-specific attention module was removed and replaced with a uniform global max-pooling operation. Consequently, all classification tasks shared the same pooled representation without learning independent attention maps for different diagnostic objectives. Additionally, the attention sparsity regularization term was disabled. The obtained results reveal a decline in classification performance when compared with the complete architecture. Without task-specific attention, the network is unable to selectively focus on anatomical regions that are most relevant for individual occlusal tasks. This limitation is particularly important for orthodontic diagnosis, where different clinical conditions may depend on distinct spatial regions of the dental arches. The model achieved accuracies of 86.65%, 69.10%, 66.02%, 58.90%, and 46.71% for sagittal left, sagittal right, anterior bite, transversal bite, and midline classification, respectively, yielding an overall accuracy of 65.28%. This highlights the effectiveness of the task-specific attention mechanism in generating discriminative feature representations and enabling the network to learn clinically meaningful spatial focus patterns for each diagnostic task.

#### 4.5.5 Analysis of the proposed network under different loss function configurations

This ablation study was conducted to evaluate the contribution of the individual loss components incorporated within the proposed training objective. The complete optimization framework combines weighted classification loss, bilateral symmetry regularization, and symmetric contact constraints to encourage both discriminative feature learning and anatomically consistent occlusal representations. To assess the effect of each component, four different loss configurations were evaluated. In the first configuration, the network was trained using only the weighted cross-entropy loss. This setting optimized the model solely based on occlusal label prediction without imposing any geometric or anatomical constraints. As a result, the network relied entirely on discriminative feature learning and did not explicitly account for structural consistency between the dental arches.

The second configuration incorporated bilateral symmetry regularization in addition to the classification loss. The bilateral term encourages the model to learn anatomically plausible representations by promoting consistency between corresponding regions of the left and right sides of the dental arches. This constraint reduces unrealistic asymmetries and improves the stability of the learned geometric features. The third configuration further introduced the symmetric contact constraint. This additional component encourages anatomically meaningful contact relationships between the maxillary and mandibular arches by enforcing realistic inter-arch alignment. The inclusion of this geometric prior allows the network to better capture occlusal interactions that are important for clinical diagnosis.

Finally, the complete loss formulation combines all loss components used in the proposed architecture. By jointly optimizing classification performance, bilateral consistency, inter-arch contact relationships, and attention sparsity, the network is guided toward learning representations that are both discriminative and anatomically coherent. The results demonstrate that progressively incorporating these constraints improves the quality of the learned feature representations and contributes to more reliable occlusal classification. Table 3 presents the detailed performance comparison obtained using the different loss configurations.

**Table 3:** Summary of the performance metrics obtained with different loss function configurations using the proposed network.

Experiment	Precision	Recall	F1-score	Accuracy
Weighted cross-entropy loss	72.74	72.67	72.68	73.10
Weighted cross-entropy + bilateral symmetry loss	73.59	73.45	73.50	74.11
Weighted cross-entropy + symmetry contact loss	79.66	78.30	78.50	78.50
Weighted cross-entropy + symmetry contact + attention sparsity loss	82.59	80.46	80.15	80.46

#### 4.5.6 Analysis of the proposed network with classical classification head

This experiment evaluates an alternative version of the proposed network in which the quantum-enhanced decision layers are replaced with fully connected classical classification layers while preserving the remaining geometric-processing modules. This configuration achieved an average accuracy of 75.00% across the evaluated tasks. However, this experiment was designed as an architectural ablation rather than a capacity-matched comparison between classical and quantum classifiers. Differences in classifier formulation and optimization therefore prevent attribution of the observed performance difference specifically to the quantum component. Accordingly, these results are presented as an exploratory evaluation of the VQC-based classification stage.

#### 4.5.7 Analysis of the proposed network

This subsection presents the performance of the complete proposed system, which integrates FRC, FKC, the mesh convolutional backbone, and hybrid quantum-classical classification heads. The mesh convolutional layers and classification heads process features obtained through cyclically invariant, rotation-aware FRC encoding and kernel-based surface-orientation correlation. The proposed network achieved accuracies of 92.31% and 87.50% for sagittal left and right relationships, respectively, while accuracies of 75.00% and 65.00% were obtained for anterior bite and midline classification. The complete system achieved an overall accuracy of 80.46% on the held-out test cohort. The system was tested on five independent random seed values: 42, 123, 256, 512, and 1,024. The obtained average performance metrics were, 78.22 ± 4.3 accuracy, 78.59 ± 5.31 precision, 76.67 ± 3.91 recall, and 77.12 ± 2.35 F1-score. Tables 4, 5 present the summary of the results obtained during the ablation studies and the class-wise performance metrics, respectively. Task-specific diagnostic distribution and classification errors across all evaluated occlusal parameters are detailed in Figure 6.

**Table 4:** Summary of the computed unweighted metrics across all occlusal tasks evaluated during the ablation studies.

Experiment	Number of parameters (M)	GFLOPS	Inference time (ms)	Sagittal left (in %)	Sagittal right (in %)	Anterior bite (in %)	Transversal bite (in %)	Accuracy of midline symmetry (in %)	Overall accuracy (in %)
Without cross-jaw interactions	30.26	0.935	80.55	62.04	49.75	54.75	73.72	56.00	59.25
Without FRC module	30.25	0.935	71.19	71.22	60.32	44.36	68.63	55.54	59.99
Without FKC module	30.24	0.935	70.79	86.79	78.94	62.71	68.45	55.33	70.44
Without Task-Attention	28.43	0.935	68.24	85.65	69.10	66.02	58.90	46.71	65.28
With classical classification head	30.26	0.935	75.51	90.00	85.00	65.00	80.00	55.00	75.00
Proposed network	30.28	0.935	155.74	92.31	87.50	75.00	82.50	65.00	80.46

**Table 5:** Class-wise performance metrics obtained from the proposed network.

Classes	Tasks	Precision	Recall	F1-score
Sagittal right	Class I	93.94	96.88	95.38
	Class II	33.33	50.00	40.00
	Class III	75.00	50.00	60.00
Sagittal left	Class I	96.97	100	98.46
	Class II	33.33	50.00	40.00
	Class III	100	60.00	75.00
Anterior bite	Deep bite	66.67	100	80.00
	Normal	72.73	53.33	61.54
	Open bite	100	77.78	87.50
	Reverse bite	100	50.00	66.67
Transversal bite	Crossbite	75.00	75.00	75.00
	Normal	85.19	88.46	86.78
	Scissor bite	100	50.00	66.67
Midlines	Centered	56.00	82.36	66.67
	Deviated	80.00	52.17	63.16

**Figure 6:**
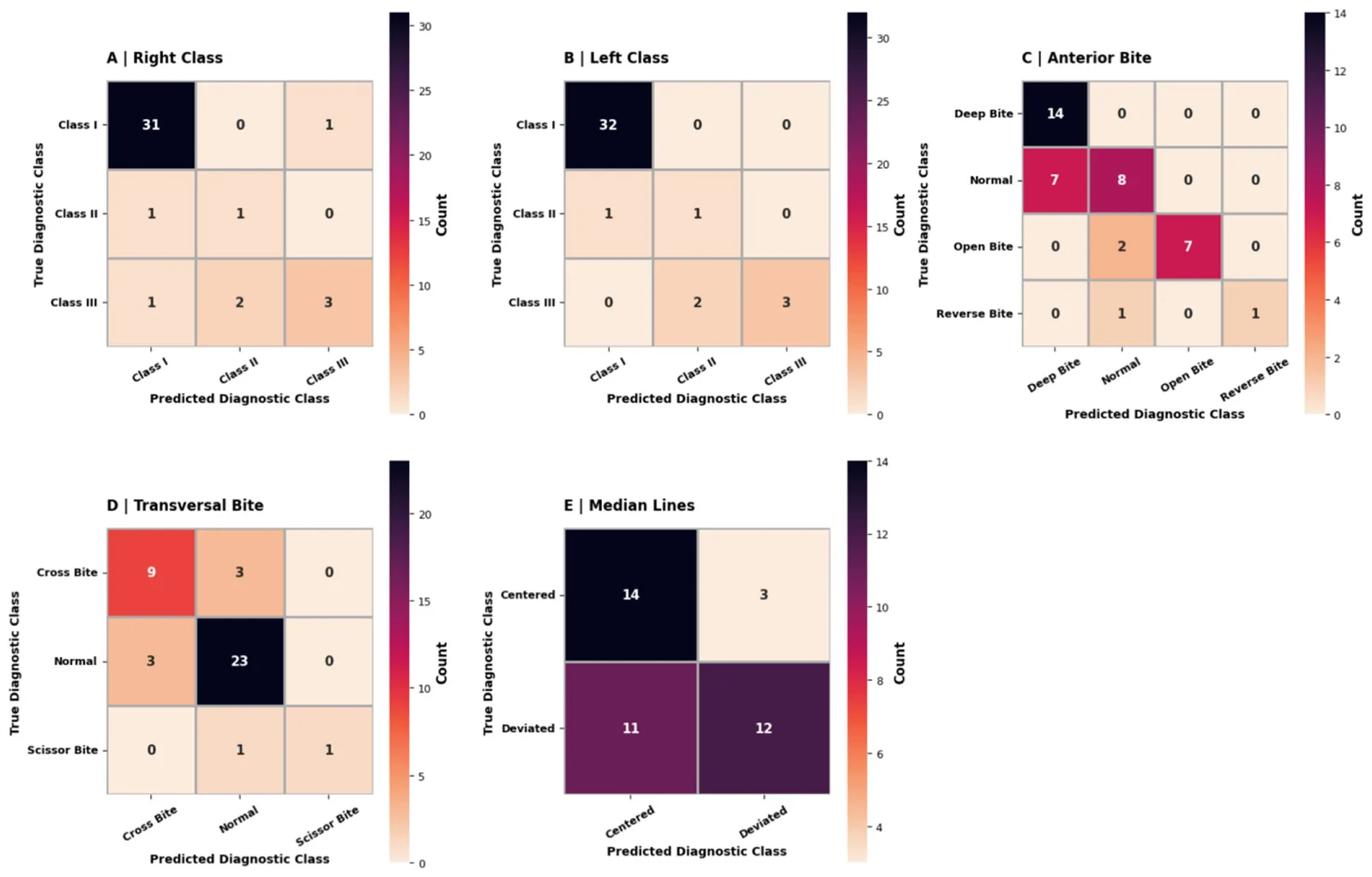
Confusion matrices of the obtained on the test cohort for: **(A)** right sagittal class, **(B)** left sagittal class, **(C)** anterior bite, **(D)** transversal bite, and **(E)** median lines. Rows indicate true labels, columns denote predicted labels, and values show patient counts.

To complement the fixed hold-out evaluation, a separate five-fold cross-validation experiment was conducted to assess the robustness of the finalized model to variations in data partitioning. This analysis was performed independently of the presented hold-out evaluation. After completion of model development, the architecture and hyperparameter configuration were fixed and were not re-tuned using the cross-validation results. For this secondary analysis, the complete dataset was partitioned into five mutually exclusive folds of approximately equal size. In each iteration, four folds were used for model training, and the remaining fold was used exclusively for evaluation. This procedure was repeated five times such that every sample served as an evaluation sample exactly once. A new model was trained for each fold, while the architecture, optimizer settings, learning rate, loss coefficients, and other selected hyperparameters were kept fixed across all five folds. No performance information from an evaluation fold was used to modify the hyperparameter configuration for that fold. The fold-wise results were subsequently aggregated to report the mean and standard deviation of each performance metric. In addition, 95% confidence intervals were estimated using Student's *t*-distribution, as given in Equation 10.


$$\begin{array}{l} CI_{95\%}=\bar{x}\pm t_{0.975,n-1}\frac{s}{\sqrt{n}} \end{array}$$ (11)

where $\bar{x}$ denotes the mean metric value across folds, *s* represents the standard deviation, *n* is the number of folds, and *t*_0.975, *n*−1_ is the corresponding critical value from the *t*-distribution. The confidence intervals provide an estimate of the statistical variability of the reported performance metrics and offer additional insight into the reliability of the proposed model. Table 6 presents the summary per-class performance metrics obtained from the proposed architecture.

**Table 6:** Summary of the performance metrics obtained by the proposed network corresponding to the mean and standard deviation along with the 95% CI computed across all five folds.

Classes	Recall	Precision	F1-score	Accuracy
Sagittal left	69.32 ± 11.74 [54.75, 83.90]	77.12 ± 10.41 [64.19, 90.05]	71.00 ± 10.09 [58.47, 83.53]	88.60 ± 7.27 [79.57, 97.63]
Sagittal right	66.03 ± 9.98 [53.64, 78.42]	68.64 ± 9.16 [57.26, 80.02]	65.4 ± 10.51 [51.92, 78.16]	82.09 ± 7.14 [73.22, 90.96]
Anterior bite	70.09 ± 10.23 [58.08, 83.50]	83.85 ± 9.66 [71.85, 95.85]	73.73 ± 9.36 [62.11, 85.36]	73.80 ± 7.46 [64.54, 83.06]
Transversal bite	72.00 ± 9.51 [60.20, 83.81]	85.65 ± 10.82 [72.22, 99.08]	76.00 ± 11.45 [61.79, 90.21]	79.00 ± 6.35 [71.12, 86.88]
Midlines	66.32 ± 9.12 [55.00, 77.64]	67.80 ± 10.51 [54.75, 80.86]	63.66 ± 10.93 [50.09, 77.23]	61.79 ± 5.21 [55.32, 68.26]

## 5 Discussion

This section discusses the performance of the proposed network and contextualizes the obtained results with previously reported findings from existing approaches. Because the referenced methods were not evaluated under an identical experimental protocol, these literature results are used only as descriptive reference benchmarks and not as controlled baseline comparisons. The section also presents the limitations observed in the current study and future works that can be incorporated to further improve the automated dental occlusion classification tasks.

### 5.1 Performance comparison with previously reported studies

This section presents the analysis of the proposed network against baseline models and existing research for occlusion classification using 3D dental mesh data. Among the existing approaches, Borghi et al. presented the highest accuracy of 71.00% using the Point-TransformerV3 network, while MeshCNN and MeshNet demonstrated nearly similar performance with accuracies of 66.25% and 65.19%, respectively. The proposed network achieved an overall classification accuracy of 80.46% on the held-out test cohort. This performance indicates the potential of the proposed geometry-aware framework for multi-task occlusal classification. However, the improved predictive performance is accompanied by increased inference time due to the additional geometric processing stages and enhanced classification heads, representing a trade-off between predictive performance and computational efficiency. It should also be noted that the existing models were not evaluated under the identical experimental configuration employed in the present study, as complete implementation and training details were not available for all approaches. Therefore, the reported results are presented as reference benchmarks rather than as a strictly controlled head-to-head comparison. Table 7 presents the summary of the comparative analysis evaluated in this study.

**Table 7:** Reference summary of the proposed network and previously reported results from existing 3D dental classification approaches.

Model	Accuracy (in %)	Inference time (in s)
PointNet++	64.2	0.09
SPUNet Borghi et al., (2025)	65.00	0.02
MeshNet	65.19	0.05
MeshCNN	66.25	0.07
PointTransformer V3 Borghi et al., (2025)	71.00	0.11
Proposed Network	80.46	1.56

The values originate from different experimental settings and do not constitute a controlled head-to-head comparison.

### 5.2 Limitations of the proposed system

The proposed system for dental occlusion classification has a few limitations that highlight the areas of future research and explorations.

- The datasets available publicly may not fully represent the variability of dental anatomies across different populations. This limits the generalization ability of the model when tested with new clinical scenarios and environments. Therefore, future studies should create bigger and more diverse datasets that include a wider range of occlusion conditions, scanner types, and demographics of patients. This can help with efficient training and the generalization capability of the automated models.
- Due to the extremely limited number of samples in certain rare occlusal classes, a small number of augmented samples derived from training instances were included in the validation subset to maintain class representation. This may introduce limited sample-level dependence between the training and validation subsets and potentially influence model-selection estimates. Importantly, augmentation was not used in the independent test subset, which contained only original samples and was used exclusively for final performance evaluation. Future studies with larger datasets should employ fully patient-independent training, validation, and test partitions prior to augmentation.
- The proposed system in this study utilizes geometry-aware components that include feature extraction, mesh-based neighborhood computation, and kernel-based correlation calculation. While these strategies help improve the performance of the system, they also increase the computational cost during inference. Hence, future works could work on optimizing newer architectures using lightweight mesh processing modules or efficient geometric sampling strategies. This helps reduce memory required and inference latency while maintaining the performance.
- A limitation of the current bilateral consistency formulation is that reflection is defined with respect to the *x* = 0 plane of the normalized mesh coordinate system rather than a midsagittal plane estimated independently for each patient. Future work could incorporate subject-specific anatomical landmark registration or learned symmetry-plane estimation before applying bilateral regularization.
- The current study employs a fixed sampling resolution of 8,192 triangular faces per mesh to preserve sufficient geometric information while maintaining computational feasibility. Future studies could further investigate different mesh sampling resolutions to assess their influence on local geometric representation and classification performance.
- The proposed architecture incorporates VQC-based hybrid quantum-classical classification heads as a proof-of-concept implementation. The current experiments do not include a systematically capacity-matched family of classical classifiers or controlled convergence analyses; therefore, the study does not establish superior representational capacity, faster convergence, or quantum advantage. In addition, all VQC experiments were conducted using quantum simulation in a classical computing environment rather than physical quantum hardware. Future work should include controlled comparisons with capacity-matched classical classifiers, systematic computational and convergence analyses, and evaluation on suitable quantum hardware when technically feasible.

## 6 Conclusion

Dental occlusion analysis plays a critical role in orthodontic diagnosis and planning of treatment. The occlusal discrepancies are closely linked to functional impairment and craniofacial abnormalities. Traditional approaches mostly rely on manual clinical inspection and intraoral scans. These are time-consuming, subjective, and highly dependent on clinician expertise. This study presents a novel method to address current limitations by utilizing a geometry-aware and quantum-enhanced DL network for the automated classification of dental occlusion by 3D mesh data. The proposed model uses a hierarchical mesh-processing backbone made up of rotation-aware feature extraction and kernel-based geometric correlation to accurately capture the local and global structural features. The FRC and FKC modules enhance the encoding of relationships at both the vertex and surface levels, thereby assisting the model in identifying subtle patterns. The architecture employs a geometry-aware learning approach along with quantum-enhanced classification heads, enabling it to simultaneously predict clinically significant occlusal attributes. The research also employs a novel composite loss mechanism that enables the model to learn effective features by focusing on anatomically important areas while addressing the class imbalance present in the dataset. On the independent held-out test cohort, the proposed network achieved an average classification accuracy of 80.46% across the evaluated occlusal tasks.

## Data Availability

Publicly available datasets were analyzed in this study. This data can be found at: https://ditto.ing.unimore.it/bits2bites/.
